# Inhibitory effects of B-cell lymphoma 2 on the vasculogenic mimicry of hypoxic human glioma cells

**DOI:** 10.3892/etm.2014.2162

**Published:** 2014-12-30

**Authors:** JIANWEN LI, YIQUAN KE, MIN HUANG, SHUYUN HUANG, YIMING LIANG

**Affiliations:** Department of Neurosurgery, Neurosurgery Institute, Key Laboratory on Brain Function Repair and Regeneration of Guangdong, Zhujiang Hospital of Southern Medical University, Guangzhou, Guangdong 510282, P.R. China

**Keywords:** B-cell lymphoma 2, hypoxia, U87 glioma cells, vasculogenic mimicry

## Abstract

The aim of this study was to investigate the mechanisms and effects of B-cell lymphoma 2 (Bcl-2) on the vasculogenic mimicry (VM) of human glioma cells. U87 cells were cultured under hypoxic conditions and then divided into four groups: Control, 3-(5-hydroxymethyl-2-furyl)-1-benzylindazole (YC-1), ABT-737 and YC-1 + ABT-737. These groups were treated with the corresponding simulators. The expression of hypoxia-inducible factor-1α (HIF-1α), matrix metalloproteinase (MMP)-2, MMP-14 and Bcl-2 in each group was determined using a reverse transcription-quantitative polymerase chain reaction and western blot analysis. Compared with that in the control group, the mRNA and protein expression of MMP-2, MMP-14 and Bcl-2 in the YC-1 and ABT-737 groups was significantly reduced. The expression of HIF-1α, however, was only significantly reduced in the YC-1 group (P<0.05). Compared with those in the YC-1 + ABT-737 group, the expression levels of the four proteins in the YC-1 and ABT-737 groups were not significantly different, with the exception of the expression of HIF-1α in the ABT-737 group, which was significantly enhanced (P<0.05). The mRNA expression levels of HIF-1α, MMP-2 and MMP-14 in the YC-1 group were significantly different from those in the ABT-737 group (P<0.01); however, no significant difference was observed in the expression of Bcl-2. In conclusion, Bcl-2 may be an important factor in the VM formation of human malignant glioma U87 cells under hypoxic conditions. Certain functions of Bcl-2 may be attributed to the HIF-1α-MMP-2-MMP-14-VM channel, whereas other functions may be independent of the channel.

## Introduction

Vasculogenic mimicry (VM) is a unique blood supply pattern present in malignant tumours. VM refers to the remodelling of tumour cells into a luminal-like structure for blood flow, although no endothelial cells are present on the inner luminal surface. The formation of VM is associated with tumour cell plasticity and the varying tumour microenvironment (1). The mechanisms underlying its formation remain unclear, but a variety of proteins and microenvironmental factors are known to contribute to VM (2–4).

The changing tumour microenvironment has a certain promoting effect on VM. Hypoxia and tumour extracellular matrix remodelling are also actively involved in VM formation (5,6). Thus, inducing hypoxic conditions can promote the biological behaviour and VM of malignant glioma (SHG-44) cells. Hypoxia-inducible factor-1α (HIF-1α)-vascular endothelial growth factor (VEGF)-Ephrin type-A receptor 2 (EphA2)-matrix metalloproteinase (MMP)-VM may be a key channel for VM formation under hypoxic conditions. In addition to HIF-1α and VEGF, which contribute to the mechanisms of VM formation, another known factor that promotes the VM formation of highly malignant glioma cells is the hypoxic condition (7,8). The 3-(5-hydroxymethyl-2-furyl)-1-benzylindazole (YC-1)-induced inhibition of HIF-1α has been found to downregulate the VM formation of glioma cells (9) under hypoxic conditions; other pathways, aside from the HIF-1α pathway, may also affect the VM formation of glioma cells (10–12).

The overexpression of the anti-apoptotic protein B-cell lymphoma 2 (Bcl-2) is considered to be an important factor in the generation, metastasis and angiogenesis of a variety of malignant tumours, including malignant glioma. Bcl-2 reportedly affects the VM formation of human melanoma cells by regulating VE-cadherin under an induced hypoxic condition (13–15). In the present study, chemical hypoxia was used to induce a hypoxic environment for human malignant glioma U87 cells, and the effects of the Bcl-2-specific inhibitor ABT-737 on the VM formation of the U87 cells was subsequently observed. The aim of the study was to explore the function and mechanism of Bcl-2 in VM formation under hypoxic conditions.

## Materials and methods

### Cell culture and establishment of model

Human malignant glioma U87 cells were obtained from the Neurosurgical Institute of Zhujiang Hospital (Guangzhou, China). Matrigel™ (Becton-Dickinson, San Jose, CA, USA) and foetal bovine serum (FBS; Gibco-BRL, Grand Island, NY, USA) were mixed at a ratio of 1:1 and were added to 24-well plates at 20 μl/well. Subsequent to solidifying the glue for 20 min at room temperature, 1×10^4^/well U87 cells were added to the plates and then incubated at 37°C for adherence. When the adherent cells reached 80% confluence, the medium was replaced by a serum-free medium. The cells were subsequently divided into four groups: Control (blank), YC-1, ABT-737 and YC-1 + ABT-737. A total of 50 μmol/l YC-1 (Cayman Chemical Co., Ann Arbor, MI, USA) and/or 50 μmol/l ABT-737 (Shanghai Han Hong Biotechnology Co., Ltd., Shanghai, China) was added to the wells accordingly. After 5 min, 100 μmol/l cobalt chloride (CoCl_2_; Sigma-Aldrich, St. Louis, MO, USA) was added to each group, and the plates were incubated for 24 h.

### Reverse transcription-quantitative polymerase chain reaction (RT-qPCR)

The mRNA expression levels of HIF-1α, MMP-2, MMP-14 and Bcl-2 in the cells of each group were analysed using RT-qPCR. Following incubation with CoCl_2_ for 24 h, the cells were harvested and dissolved in TRIzol^®^ (Invitrogen Life Technologies, Carlsbad, CA, USA) for total RNA extraction. The isolated RNA was applied as a template to an RT reaction using an RT-PCR kit (Promega Corporation, Madison, WI, USA). The qPCR reaction was performed using an Mx3000P qPCR system (Agilent Technologies, Waldbronn, Germany) for 3 min at 95°C and for 39 cycles of 10 sec at 95°C, 10 sec at 55°C and 30 sec at 72°C. Gene-specific primers for human HIF-1α, MMP-2, MMP-14 and Bcl-2 were designed by Sangon Biotech Co., Ltd. (Shanghai, China), as shown in Table I.

### Western blot analysis

The protein extraction reagent and the extraction method used for the extraction of total protein from the cells of each group were as described previously (2). The proteins were separated by 8% sodium dodecyl sulphate-polyacrylamide gel electrophoresis and transferred onto polyvinylidene difluoride membranes for immunoblot analysis. Subsequent to blocking with 5% skimmed milk powder for 2 h at room temperature, the membranes were incubated with monoclonal rabbit anti-human HIF-1α (ab51608; 1:500; Abcam, Cambridge, UK), mouse monoclonal anti-human MMP-2 (sc-13594; 1:500 Santa Cruz Biotechnology, Inc., Santa Cruz, CA, USA), polyclonal rabbit anti-human MMP-14 (ab109745, 1:500; Abcam), polyclonal rabbit anti-human Bcl-2 (sc-492-G; 1:500; Santa Cruz Biotechnology, Inc.) and GAPDH (ab14247; 1:3,000; Abcam) antibodies at 4°C overnight. The membranes were then incubated with peroxidase-conjugated secondary antibodies (EMD Millipore, Billerica, MA, USA) for 1 h at room temperature. The specific protein bands on the membranes were visualised by the enhanced chemiluminescence method and analysed using the Tanon 3500 (Tanon 3500R) Gel Imaging System (Jinglai Laboratory Equipment Co., Ltd., Shanghai, China). The absorbance of each band was measured, and the ratio of each target band to GAPDH was used to show the expression of each target protein.

### Statistical analysis

Data are shown as the mean ± standard deviation and were analysed using SPSS version 11.5 software (SPSS, Inc., Chicago, IL, USA). Comparisons between two groups were performed using the Student’s t-test. P<0.05 was considered to indicate a statistically significant difference.

## Results

### mRNA expression of HIF-1α, MMP-2, MMP-14 and Bcl-2

**Figure 1:**
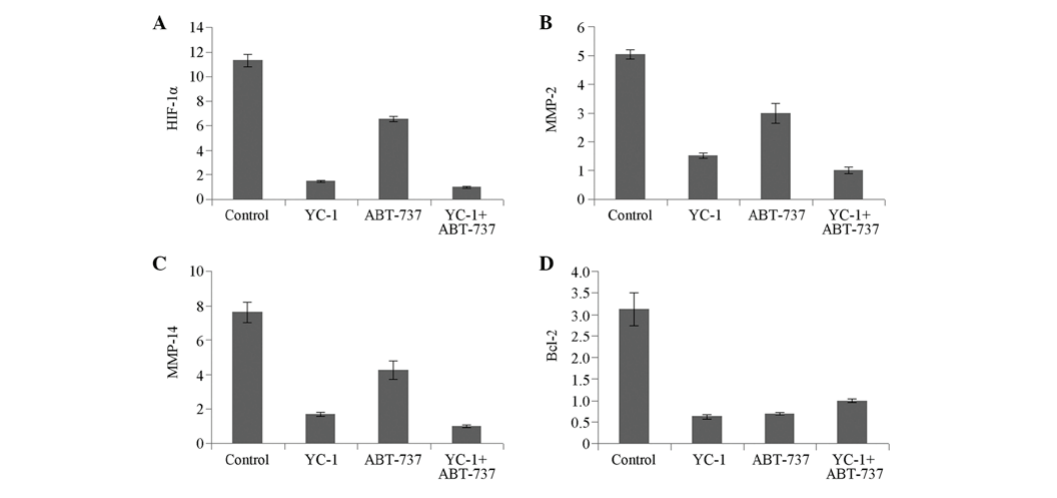
mRNA expression of (A) HIF-1α, (B) MMP-2, (C) MMP-14 and (D) Bcl-2 in U87 cells. The cells were either left blank or treated with 50 μmol/l YC-1 and/or ABT-737. MMP, matrix metalloproteinase; YC-1, 3-(5-hydroxymethyl-2-furyl)-1-benzylindazole; HIF-1α, hypoxia-inducible factor-1α; Bcl-2, B-cell lymphoma 2.

Compared with that in the control group, the mRNA expression of HIF-1α was downregulated significantly in the YC-1 group (P<0.05); this downregulation was not significant in the ABT-737 group. By contrast, the mRNA expression levels of MMP-2, MMP-14 and Bcl-2 were all significantly reduced in the YC-1 and ABT-737 groups (P<0.05) (Fig. 1).

Compared with that in the YC-1 + ABT-737 group, the mRNA expression of HIF-1α was increased significantly in the ABT-737 group (P<0.05) but increased slightly in the YC-1 group, without significance (P>0.05). The mRNA expression of MMP-2 and MMP-14 showed an increasing trend but that of Bcl-2 showed a decreasing trend in the YC-1 and ABT-737 groups compared with that in the YC-1 + ABT-737 group, although no significant differences were observed (P>0.05) (Fig. 1). The mRNA expression levels of HIF-1α, MMP-2 and MMP-14 in the YC-1 group were significantly different from those in the ABT-737 group (P<0.01) (Fig. 1A–C); however, no statistically significant difference was observed in the mRNA expression of Bcl-2 (Fig. 1D).

### Protein expression of HIF-1α, MMP-2, MMP-14 and Bcl-2

**Figure 2:**
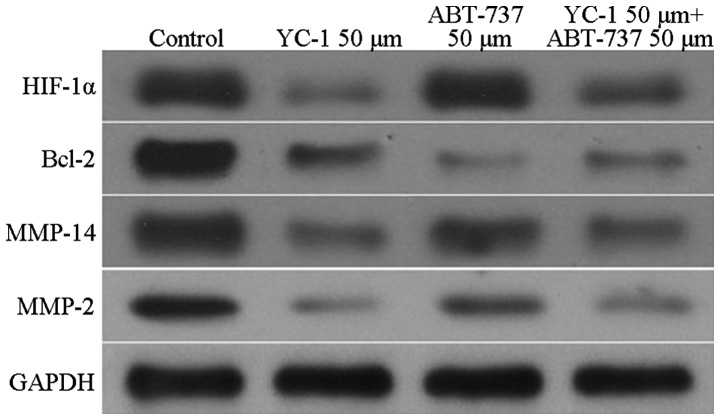
Protein expression of HIF-1α, MMP-2, MMP-14 and Bcl-2 in U87 cells. The cells were either left blank or treated with 50 μmol/l YC-1 and/or ABT-737. MMP, matrix metalloproteinase; YC-1, 3-(5-hydroxymethyl-2-furyl)-1-benzylindazole; HIF-1α, hypoxia-inducible factor-1α; Bcl-2, B-cell lymphoma 2.

The expression levels of HIF-1α, MMP-2, MMP-14 and Bcl-2 in the U87 cells underwent changes that were similar to those observed for the mRNA expression. Compared with the HIF-1α protein expression in the control group, the expression of the U87 cells in the YC-1 group was significantly decreased (P<0.05). By contrast, the expression of HIF-1α in the U87 cells in the ABT-737 group showed no significant reduction. In the YC-1 and ABT-737 groups, the expression levels of MMP-2, MMP-14 and Bcl-2 were all significantly reduced compared with those in the control group (P<0.05) (Fig. 2). Furthermore, compared with HIF-1α protein expression in the YC-1 + ABT-737 group, the expression was increased significantly in the ABT-737 group (P<0.05) but not in the YC-1 group. The expression levels of MMP-2, MMP-14 and Bcl-2 showed no significant differences between the YC-1 + ABT-737 group and the YC-1 and ABT-737 groups (P>0.05, Fig. 2).

## Discussion

In 1999, Maniotis *et al* (1) found a special microcirculatory system in the highly aggressive uveal melanoma, in which the channels were covered not with endothelial cells but with a layer of tumour cells. Blood flow was noted within the lumen. Occasionally, a layer of periodic acid-Schiff-positive stromal membrane separating the tumour cells from blood cells was observed; this mechanism was eventually termed VM by Maniotis *et al* (1). VM was then gradually found in ovarian (16), breast (17), liver (18) and stomach (19) cancer. Further experiments demonstrated that VM only occurs in extremely malignant tumours (20) and that hypoxia can promote its formation in these tumours (15,20,21). VM has also been found in malignant glioblastomas, and the induced hypoxic condition has been found to affect the VM formation of gliomas (22). Furthermore, hypoxia can promote the VM generation of low-grade malignant glioma cells. The HIF-1α and VEGF-EphA2-MMP-VM channels are reportedly key channels for VM formation under hypoxic conditions. VM has also been found among the three-dimensional culture of U-251 human glial cells. In recent studies, hypoxia was confirmed to promote the VM formation of glioma cells, and the induced HIF-1α and VEGF-A expression was suggested a mechanism involved in this process (10,23,24).

YC-1 has superior anti-angiogenic and tumour therapeutic activities. Wohlfart *et al* (21) found that YC-1 could promote nitric oxide (NO) synthesis in foetal bovine aortic endothelial cells in a concentration-dependent manner; however, NO inhibits the stability and transcriptional activity of HIF-1α under hypoxic conditions. NO has also been shown to have inhibitory effects on VEGF and GPI genes in human pancreatic cancer cells under hypoxic conditions (8). Gariboldi *et al* (9) reported that YC-1 exerts inhibitory effects on the VM formation of U87 cells. Few reports, however, have focused on the VM formation mechanisms of human glioma U87 cells under the hypoxic condition (25,26).

The mechanisms underlying VM formation remain unclear. Bcl-2 is currently considered to be an important factor for the generation, metastasis and angiogenesis of malignant tumours (5,9). In conditions of induced hypoxia, Bcl-2 can affect the VM formation of human melanoma by regulating the expression of VE-cadherin (27). An investigation into the possible effect of Bcl-2 on VM formation inside hypoxic U87 cells is likely to aid the understanding of VM formation mechanisms inside hypoxic U87 cells.

In CoCl_2_-induced hypoxia, cobalt can replace the chelated iron in haemoglobin and damage the oxygen sensation of cells. CoCl_2_ has stimulated a hypoxic environment in cultured cells that is strongly comparable to hypoxia *in vivo*. Since glioblastoma cells are in an induced hypoxic microenvironment, tumour cells fuse to form VM and then provide nutrition for the tumour. Thus, during the inhibition of tumour blood vessels, understanding the channels for VM and applying comprehensive treatment programs can cut off the tumour blood supply satisfactorily and consequently cure tumours (28–30).

In the present study, U87 cells were treated with the specific inhibitors of Bcl-2 (ABT-737) and HIF-1α (YC-1). The cells were then cultured under an induced hypoxic condition. Subsequent to the cells being cultured with CoCl_2_ for 24 h, the expression of HIF-1α, MMP-2, MMP-14 and Bcl-2 in the U87 cells was determined. In the current study, it was observed that the expression of HIF-1α in the U87 cells in the YC-1 group was significantly reduced (P<0.05) but the ABT-737 group exhibited no significant reduction. In the YC-1 + ABT-737 group, the expression levels of MMP-2, MMP-14 and Bcl-2 were all significantly reduced compared with those in the control group (P<0.05). No significant differences were observed in the expression of Bcl-2 among the YC-1 + ABT-737, ABT-737 and YC-1 groups. This finding indicates that Bcl-2 does not play a role in VM via the HIF-1α pathway. HIF-1α-MMP-2-MMP-14-VM has previously been confirmed as a traditional pathway (2,5). Through a different pathway, the Bcl-2 inhibitor influenced the MMP-2 and MMP-14 expression levels, but not HIF-1α expression levels, in hypoxic U87 cells significantly. This finding indicates that Bcl-2 inhibitors were not involved in VM formation via the traditional pathway. In conclusion, Bcl-2 may be an important factor in the VM formation of human malignant glioma U87 cells under hypoxic conditions. Bcl-2 may exert its effects both through the HIF-1α-MMP-2-MMP-14-VM channel and through other means that are independent of this channel. This finding indicates that hypoxia affects VM formation not simply by influencing the expression of HIF-1α. Further studies on the mechanisms underlying the VM formation of glioma cells under hypoxic conditions should therefore be carried out.

## Figures and Tables

**Table I:** Gene-specific primer pairs used in this study.

Genes	Forward primer (5′-3′)	Reverse primer (5′-3′)
MMP-2	CGCATCTGGGGCTTTAAACAT	CCATTAGCGCCTCCATCGTA
MMP-14	CCGATGTGGTGTTCCAGACA	TCGTATGTGGCATACTCGCC
HIF-1α	GCGCGAACGACAAGAAAAAGA	GTGGCAACTGATGAGCAAGC
Bcl-2	CTTTGAGTTCGGTGGGGTCA	GGGCCGTACAGTTCCACAAA
GAPDH	AAGGTGAAGGTCGGAGTCAA	AATGAAGGGGTCATTGATGG

MMP, matrix metalloproteinase; HIF-1α, hypoxia-inducible factor-1α; Bcl-2, B-cell lymphoma-2.
